# Impaired Cognitive Function and Altered Hippocampal Synapse Morphology in Mice Lacking *Lrrtm1*, a Gene Associated with Schizophrenia

**DOI:** 10.1371/journal.pone.0022716

**Published:** 2011-07-27

**Authors:** Noriko Takashima, Yuri S. Odaka, Kazuto Sakoori, Takumi Akagi, Tsutomu Hashikawa, Naoko Morimura, Kazuyuki Yamada, Jun Aruga

**Affiliations:** 1 Laboratory for Behavioral and Developmental Disorders, RIKEN Brain Science Institute (BSI), Wako-shi, Saitama, Japan; 2 Support Unit for Neuromorphological Analysis, RIKEN Brain Science Institute (BSI), Wako-shi, Saitama, Japan; 3 Support Unit for Animal Experiments, RIKEN Brain Science Institute (BSI), Wako-shi, Saitama, Japan; Louisiana State University Health Sciences Center, United States of America

## Abstract

Recent genetic linkage analysis has shown that *LRRTM1* (*Leucine rich repeat transmembrane neuronal 1*) is associated with schizophrenia. Here, we characterized *Lrrtm1* knockout mice behaviorally and morphologically. Systematic behavioral analysis revealed reduced locomotor activity in the early dark phase, altered behavioral responses to novel environments (open-field box, light-dark box, elevated plus maze, and hole board), avoidance of approach to large inanimate objects, social discrimination deficit, and spatial memory deficit. Upon administration of the NMDA receptor antagonist MK-801, *Lrrtm1* knockout mice showed both locomotive activities in the open-field box and responses to the inanimate object that were distinct from those of wild-type mice, suggesting that altered glutamatergic transmission underlay the behavioral abnormalities. Furthermore, administration of a selective serotonin reuptake inhibitor (fluoxetine) rescued the abnormality in the elevated plus maze. Morphologically, the brains of *Lrrtm1* knockout mice showed reduction in total hippocampus size and reduced synaptic density. The hippocampal synapses were characterized by elongated spines and diffusely distributed synaptic vesicles, indicating the role of *Lrrtm1* in maintaining synaptic integrity. Although the pharmacobehavioral phenotype was not entirely characteristic of those of schizophrenia model animals, the impaired cognitive function may warrant the further study of *LRRTM1* in relevance to schizophrenia.

## Introduction

Elucidation of the genetic factors involved in schizophrenia is one of the major challenges in current neurobiology [1]-[6]. *LRRTM1* (*Leucine rich repeat transmembrane neuronal 1, OMIM 610867*) is an emerging candidate gene for schizophrenia. A three-marker haplotype upstream of *LRRTM1* on 2p12 is associated with schizophrenia/schizoaffective disorder when inherited paternally [7], [8].

In biological terms, *LRRTM1* (humans) and *Lrrtm1* (mice) encode a single-membrane-spanning transmembrane protein with a leucine-rich repeat domain in its N-terminal side, and they are predominantly expressed in the nervous systems of humans and mice, respectively [7], [9]. Tagged-rat Lrrtm1 protein is localized in the excitatory synapses of cultured hippocampal neurons and shows synaptogenic activity in neuron/fibroblast coculture assay [10]. Furthermore, the distribution of vesicular glutamate transporter (VGLUT1) is altered in *Lrrtm1* ^–/–^ mice [10]. These results raise the possibility that *Lrrtm1* is essential for higher brain function in mammals, but this possibility has not been addressed to date.

Schizophrenia is a relatively common mental disorder that affects 1% of the population worldwide. The disease is characterized by positive symptoms (delusions and hallucinations), negative symptoms (affective flattening and social withdrawal), and cognitive dysfunction (deficits in working memory, attention, processing speed, and executive function) [1], [2]. Morphologically, there are abnormalities of the brain that are hallmarks of schizophrenia, such as enlarged ventricles, reduced hippocampal volume, dendritic changes in the pyramidal neurons, and alteration of specific subtypes of interneurons [11]–[14]. Several model mice that partially mimic these behavioral and morphological signs have been developed, contributing to our understanding of the pathophysiology of schizophrenia [3]–[6], [15], [16].

Here, we investigated the behavioral properties of *Lrrtm1* knockout (KO) mice. These mice showed deficits in behavioral responses to stressful situations and novel objects, together with spatial memory and social discrimination deficits. In addition, we clarified some of the morphological abnormalities of the mutant's hippocampus; these deficits may be related to the behavioral abnormalities found.

## Results

### Generation of *Lrrtm1*-null mutant mice

**Figure 1:**
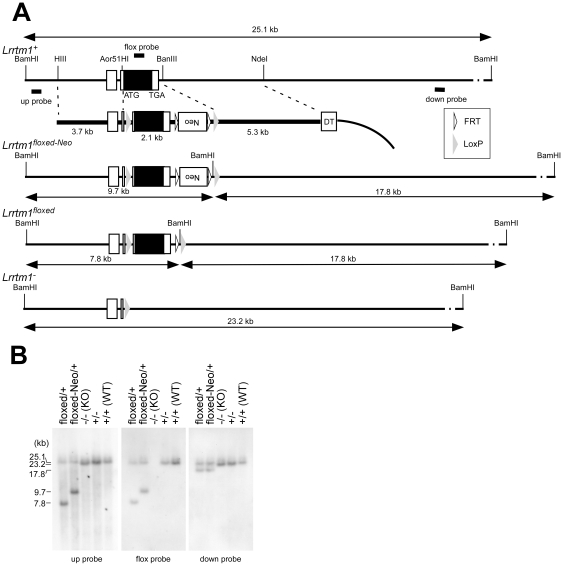
Targeted disruption of the *Lrrtm1* gene. (A) Structures of the *Lrrtm1* genomic locus, targeting vector, and mutated allele. Locations of the 5′ and 3′ probes for Southern blotting are shown. Solid box, protein coding region of the exons; open box, untranslated region of the exons; gray triangle, loxP site; open triangle, FRT site; DT, diphtheria toxin A; Neo, neomycin-resistance gene cassette; ATG, initiation codon; TGA, termination codon. Lines with double arrowheads indicate restriction fragment lengths. (B) Confirmation of homologous recombination of the mutant alleles by Southern blot. *Bam*HI-digested genomic DNA was hybridized with genomic fragments that corresponded to the genomic sequences of 5′ and 3′ outside the targeting vector (up probe and down probe, respectively) and an Lrrtm1 protein-coding region (flox probe).

We generated an *Lrrtm1* null-type mutation (*Lrrtm1* ^–^) by homologous recombination in ES cells (Figure 1). Mating between heterozygotes (*Lrrtm1^+/^* ^−^) generated homozygotes (*Lrrtm1^–/^* ^–^, *Lrrtm1* KO) in an expected Mendelian ratio when examined at weaning (+/+, 23%, +/–, 50%; –/–, 27%; n = 205). The mice grew with normal body weight without any abnormalities in terms of external appearance (data not shown). They showed no obvious ataxic movements in observations during breeding and colony maintenance procedures.

### *Lrrtm1*-deficient mice are impaired in adaptive behaviors to environmental changes

**Figure 2:**
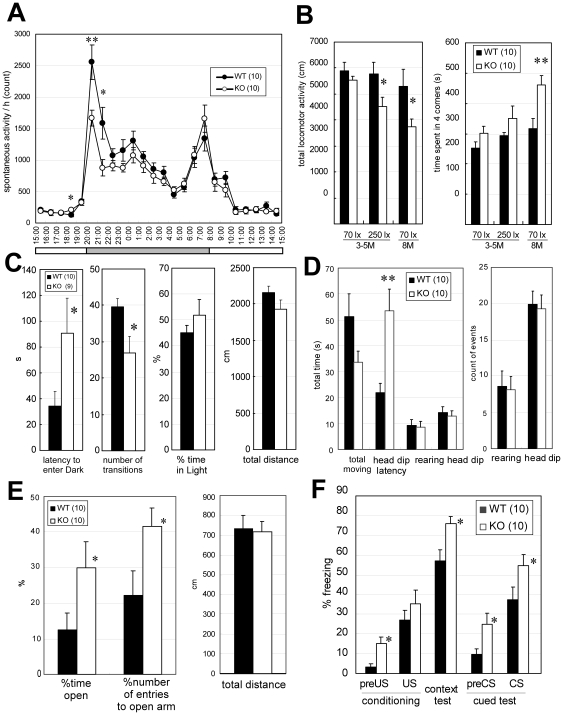
*Lrrtm1* KO mice show adaptive behavior abnormalities. (A) Home-cage activities. The circadian profile of the locomotor activity (bin  = 1 h) was first determined for each mouse. Then the mean and SEM of the locomotor activities per 1 h were calculated for each genotype. Statistical analysis was performed against the mean values for each mouse. The horizontal bar below the graph indicates the light–dark cycle (gray, dark phase; white, light phase). Values are presented as means ±SEM. * *P*<0.05; ** *P*<0.01. (B) OF test. (left) The locomotor activity indicates the total distance traveled (cm) in the test period. (right) Time spent in the four corner squares of a 5×5 subdivision of the field. Young adult mice (3 to 5 months (M)) that were new to the OF apparatus were subjected to the test at two different illuminances (70 lx or 250 lx, 3–5 M). Eight-month-old mice that had experienced several behavioral tests were also tested at 70 lx illuminance (70 lx, 8 M). Values are presented as means ±SEM. * *P*<0.05. (C) light–dark box transition test. Total distance traveled, % of time spent in the light box, number of transitions between the light and dark boxes, and the first latency period before entering the dark box are indicated as means ±SEM. * *P*<0.05. (D) Hole board test. Total moving time (s), latency until head-dipping (s), number of head-dips, duration of head-dips (s), duration of rearing (s), and number of rearings are indicated as means ±SEM. ** *P*<0.01. (E) Elevated plus maze test. Total distance traveled, % time spent in the open arms, and % of entries to the open arms were measured. Values are presented as means ±SEM. * *P*<0.05 in U-test. (F) Fear-conditioning test. In both contextual and cued (conditional) tests, *Lrrtm1* KO mice exhibited significantly greater freezing responses than WT mice. * *P*<0.05; U-test. US, unconditioned stimulus; CS, conditioned stimulus. The numbers in parentheses in the key boxes indicate those of WT and KO mice used in each experiment (common to all figures).

We first measured spontaneous activities in the home cages and in open-field (OF) boxes. Over 7 consecutive days of observation in a new cage, *Lrrtm1* KO mice showed 40% to 50% less activity than wild-type (WT, *Lrrtm1^+/^* ^+^) mice in the initial 2 h of the dark (night) phase (20:00 to 21:00, *P* = 0.0085; 21:00 to 22:00, *P* = 0.022) (Figure 2A), although mean activity did not differ significantly (F(1,18)  = 2.46, *P* = 0.13). In the 15-min observation period in the OF box (Figure 2B), young adult KO mice (3 to 5 months old) showed significantly less locomotor activity than WT mice under bright illumination (250 lx) (*P* = 0.046) but not so under darker conditions (*P* = 0.28) (70 lx). Eight-month-old KO mice that had experienced several behavioral tests showed less locomotor activity (*P* = 0.044) than WT mice under 70 lx, as well as a significant preference to stay in the corners of the OF box (*P* = 0.0053) (Figure 2B). Thus, spontaneous activities differed between WT and KO mice in these two situations of environmental change.

In the light–dark box transition (LD) test (Figure 2C) mice were first placed in the light side of the box. WT mice moved to the dark box after a short while (mean 34 s), but the latency of the transition time in KO mice was much longer (mean 90 s, *P* = 0.035). In addition, the total number of transitions made by KO mice during the 10-min observation period was significantly lower than that by WT mice (*P* = 0.034). The total time spent in the light side of the box and the total distance traveled did not differ significantly between the two genotypes. Similar abnormalities were found in the hole board (HB) test (Figure 2D) [17]. In this test, mice were placed in an OF-like apparatus with four holes (3 cm diameter) on the floor (50 cm×50 cm), and their behaviors were observed for 5 min. *Lrrtm1* KO mice showed a prolonged mean latency to the time of first head-dipping behavior (*P* = 0.0042 by Welch's *t*-test), whereas the total duration and number of head-dipping behaviors were comparable with those in WT mice. There were no differences in terms of the duration and number of rearing behaviors. The LD and HB tests results suggested that the expected behavior responses in the novel environments were impaired in KO mice.

KO mice also showed behavioral abnormalities in stressful situations. In the elevated plus maze (EPM) test (Figure 2E), KO mice spent significantly more time on the open arms (U = 23, *P* = 0.041) and entered the open arms more frequently (U = 23, *P* = 0.041) than did WT mice. The total distance traveled by KO mice was comparable to that by WT mice. Although the increased time spent in the open arms and entering the open arms could be interpreted as indicating a decrease in anxiety-like tendencies, this seemed not to be the case. Because KO mice tended to freeze more frequently than WT at 1-m-high, 15-cm-diameter circle platform [freezing time (s, means ±SEM) in total 300 s observation: WT, 135±13.8 (n = 10); KO, 173±20.1 (n = 10); U = 34, *P* = 0.082], and we observed a significant increase in the number of feces in the EPM test [WT, 0.50±0.27 (n = 10); KO, 2.0±0.39 (n = 10); U = 18, *P* = 0.0094]. Accordingly, in the fear-conditioning (FC) test, KO mice showed greater freezing responses in conditioning (pre-US [unconditioned stimulus], U = 18, *P* = 0.013), a context test (U = 19.5, *P* = 0.021), and a cue test (pre-CS [conditioned stimulus], U = 23, *P* = 0.041; CS, U = 23.5, *P* = 0.045) (Figure 2F). Although our initial attempt was to assess fear memory by the FC test, this was hard to assess owing the consistently higher freezing responses.

In sum, the results of the LD, HB, EPM, and FC tests revealed behavioral deficits of *Lrrtm1* KO mice under stressful situations that urged the mice to execute adaptive responses.

### Differential responses to both inanimate and animate objects are observed in *Lrrtm1* KO

**Figure 3:**
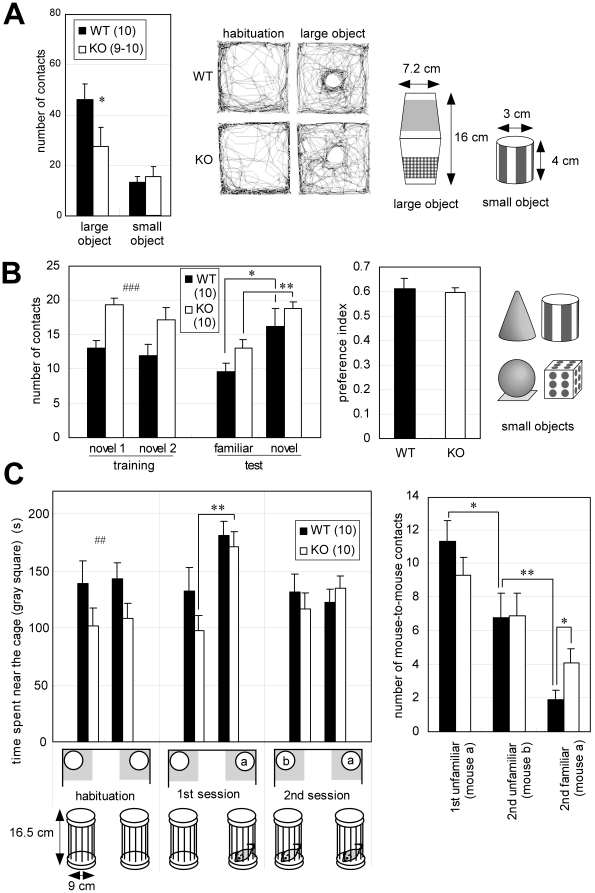
Approach to inanimate and animate objects. (A) Behavioral tests of approach to inanimate objects in the OF. The mice were first placed in the OF box without the object (habituation), then placed again in the OF with the large or small object (*right*). Approach was measured by the numbers of direct contacts with the large or small object (*left*). The traces are representative ones of WT and KO mice during the habituation and the test session with the large object (*middle*; results are given for a pair that showed comparable trace patterns in habituation). Values are presented as means ±SEM. * *P*<0.05. (B) Novel object recognition test using four kinds of small object (*right*). *novel1* and *novel2* indicate that the same kinds of objects were placed in the left and right corners, respectively, of the cages in the training session. *familiar* and *novel* indicate respectively that the object was unchanged (*novel1*) and that a new, differently shaped object was added in place of *novel2* in the test session. A novel object *preference index* was calculated as follows: contacts with *novel* / total contacts with *novel* and *familiar*. Values are presented as means ±SEM. * *P*<0.05; ** *P*<0.01. ^###^ *P*<0.001 (total contacts, comparison between WT and KO). (C) Social discrimination test. Approach to the cages was measured by the time spent in the rectangular region (indicated as gray squares below graph, 17.7 cm×17.7 cm) that included the cage (left). Mouse-to-mouse contacts (right). Values are presented as means ±SEM. * *P*<0.05; ** *P*<0.01. ^##^ *P*<0.01 (total stayed time between WT and KO).

To further clarify the adaptive behavior abnormalities, we investigated the mice's responses to inanimate and animate objects. We used two different-sized inanimate objects. The larger one was 16 cm high, with a cylindrical shape and the smaller one was 4 cm high, with a column shape (Figure 3A, far right panel). The objects was placed in the center of the OF test box (50 cm×50 cm). The number of contacts with the object were measured (Figure 3A). *Lrrtm1* KO mice contacted the large object significantly less frequently (*P* = 0.033) than did WT mice. This result was also supported by trace pattern abnormality (Figure 3A, middle). In contrast, when small objects were placed in the OF box, KO and WT mice contacted the object equally (Figure 3A); this was significantly different from the case with the large object (*P* = 0.028, F(1,35) = 5.4, two-way ANOVA for genotype-object size interaction).

To test whether the perception of “novelty” was altered in *Lrrtm1* KO mice, we also used the small objects 3–4 cm high cone, sphere, and cube in addition to the column (Figure 3B, far right panel). The surfaces of these objects were differentially labeled with black or gray on a white background. In a home cage (17 cm×28 cm×12 cm [H]), contact with the small objects by KO mice was significantly more frequent than by WT mice (Figure 3B, *training*, *P* = 0.00024), indicating that the approach to inanimate objects was context dependent. In the novel object recognition (NOR) test, two identical objects were first placed in the cage. After 15 min of exposure to the objects (Figure 3B, *training*), one object was replaced with a new one that differed in terms of shape and surface pattern. In the following 15 min, the mice were exposed to both the new, unfamiliar object and the familiar object (Figure 3B, *test*). The contacts with each object were counted in both sessions. In the NOR *test* session, both WT and *Lrrtm1* KO mice showed significantly more frequent contact with the novel object (WT, *P* = 0.033; KO, *P* = 0.0022) than with the familiar one, and the novel object preference indices of the WT and KO mice were almost the same (Figure 3B, *right*). The result suggested that an altered preference for “novelty” might not explain the above-described behavioral abnormalities.

To examine responses to animate objects, we performed a social discrimination (SD) test (Figure 3C). In this test, the mice were first habituated to empty cages (16.5 cm high, cylindrical) placed in two corners of the OF box. Before the first session, one empty cage was replaced with a cage containing a mouse. After the first session of 15 min, a new (unfamiliar) caged mouse and the familiar caged mouse were presented to the test mouse for 15 min as the second session. The results were quantified as the time spent near each cage and as the number of direct contacts through the wire slits. First, we noticed that *Lrrtm1* KO mice avoided approaching the empty cages in the habituation session (*P* = 0.0084). This result seemed consistent with the avoidance of the large object (Figure 3A). However, the empty-cage-avoidance tendency disappeared in the second and third exposures to the empty cages in a control experiment (data not shown). KO mice showed a clear preference for the caged animals in the first session, in comparison with the empty cages (*P* = 0.0023). In the second session, WT mice contacted the unfamiliar mice 3.6 times more frequently than the familiar mice. This preference was not as strong (1.7 times) in *Lrrtm1* KO mice; in fact, they contacted the familiar mice twice as frequently as did WT mice (Figure 3C) (*P* = 0.041). The results suggested a deficit in social recognition performance in *Lrrtm1* KO mice.

### Spatial memory deficits and other behavioral abnormalities in *Lrrtm1* KO mice

**Figure 4:**
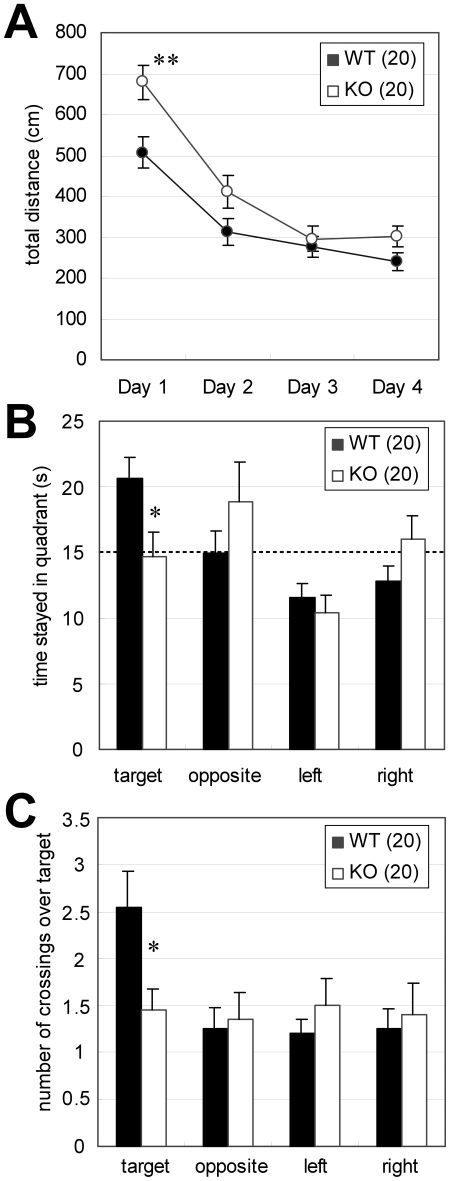
Spatial memory deficits in *Lrrtm1* KO mice. (A) Morris water maze training session. The total distance swum before reaching the target was significantly greater in KO mice than in WT mice on the first day, whereas it was comparable to that in WT mice on the second to fourth days. Values are presented as means ±SEM. * *P*<0.05; ** *P*<0.01. (B, C) Morris water maze probe test. Both the time spent in the target quadrant (B) and the number of crossings over the targets (C) were lower in *Lrrtm1* KO mice than in WT mice. Dotted line indicates the chance level. Values are presented as means ±SEM. * *P*<0.05 in U-test.

Having shown that adaptive behavior abnormalities were present in *Lrrtm1* KO mice, we then investigated other behavioral features. The Morris water maze (MWM) test is a useful common platform for assessing spatial memory. We performed 4 days of training sessions consisting of six trials per day. First, KO mice swam significantly farther than the WT mice on the first day of the 4 consecutive training days (*P* = 0.0041) (Figure 4A). In light of the above-mentioned results, we considered that this result reflected a delayed response to novel environments. In probe tests performed on the fifth day, the *Lrrtm1* KO mice showed significantly poorer performance, both in stay time in the target quadrant (U = 109.5, *P* = 0.014) and in crossing the position of the target platform (U = 128.5, *P* = 0.048) (Figure 4B and 4C). The results indicated that the KO mice had a spatial memory deficit. Notably, KO mice showed unusual behaviors during the MWM test, such as frequent dives to reach the platform (7 out of 10 KO mice but none of the WT mice showed diving behavior) and frequent rearing after reaching the platform (5 out of 10 KO mice but none of the WT mice showed rearing).

There were no significant differences between the two groups in the other behavioral tests (Table S1).

### Morphological changes in the *Lrrtm1* KO hippocampus

**Figure 5:**
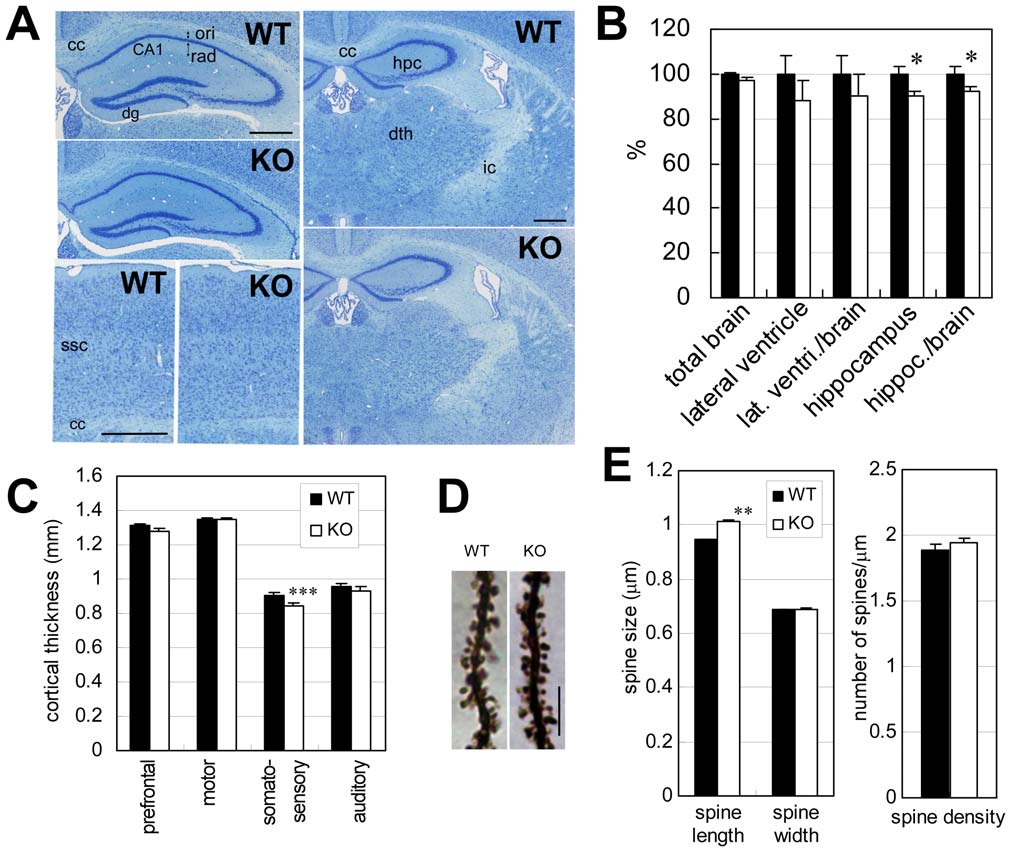
Morphological abnormalities in the *Lrrtm1* KO brain. (A) Histological examination of the hippocampus, thalamus, and cerebral cortex from WT and *Lrrtm1* KO mice. Scale bar, 0.5 mm. CA1, hippocampal CA1 area; cc, corpus callosum; dg, dentate gyrus; dth, dorsal thalamic nuclei; hpc, hippocampus; ic, internal capsule; ori, stratum oriens; rad, stratum radiatum; ssc, somatosensory cortex. (B) Volumetric analysis using MRI. Ten pairs of 36-week-old WT and *Lrrtm1* KO mice were subjected to in vivo analysis. (C) Thickness of cerebral cortices. Histological sections through prefrontal cortex, motor cortex, somatosensory cortex, and auditory cortex were subjected to morphometric analysis. (D) Spine morphology. Golgi-impregnation staining of hippocampal CA1 pyramidal neuron dendrites. Scale bar, 5 µm. (E) Length and width of spines (left) and number of spines (right) are quantified from secondary or tertiary dendrite segments (more than 20 µm; WT, 58 from 5 mice; KO, 53 from 4 mice). Mean values for each segment were analyzed. Black bars, WT; open bars, KO. Values are presented as means ±SEM. * *P*<0.05; ** *P*<0.01; *** *P*<0.001.

Histological examination of *Lrrtm1* KO adult brain sections stained with cresyl violet did not reveal any strong qualitative architectural abnormalities (Figure 5A). However, when we performed MRI scanning to search for volume changes, the *Lrrtm1* KO brain showed significant reductions in hippocampus volume (*P* = 0.029) and in the volume of the hippocampus relative to the total brain volume (*P* = 0.046) (Figure 5B). Measurement of cortical thickness indicated that there was a slight (6.6%) but significant reduction (*P*<0.001) in the thickness of the somatosensory cortex (Figure 5C).

**Figure 6:**
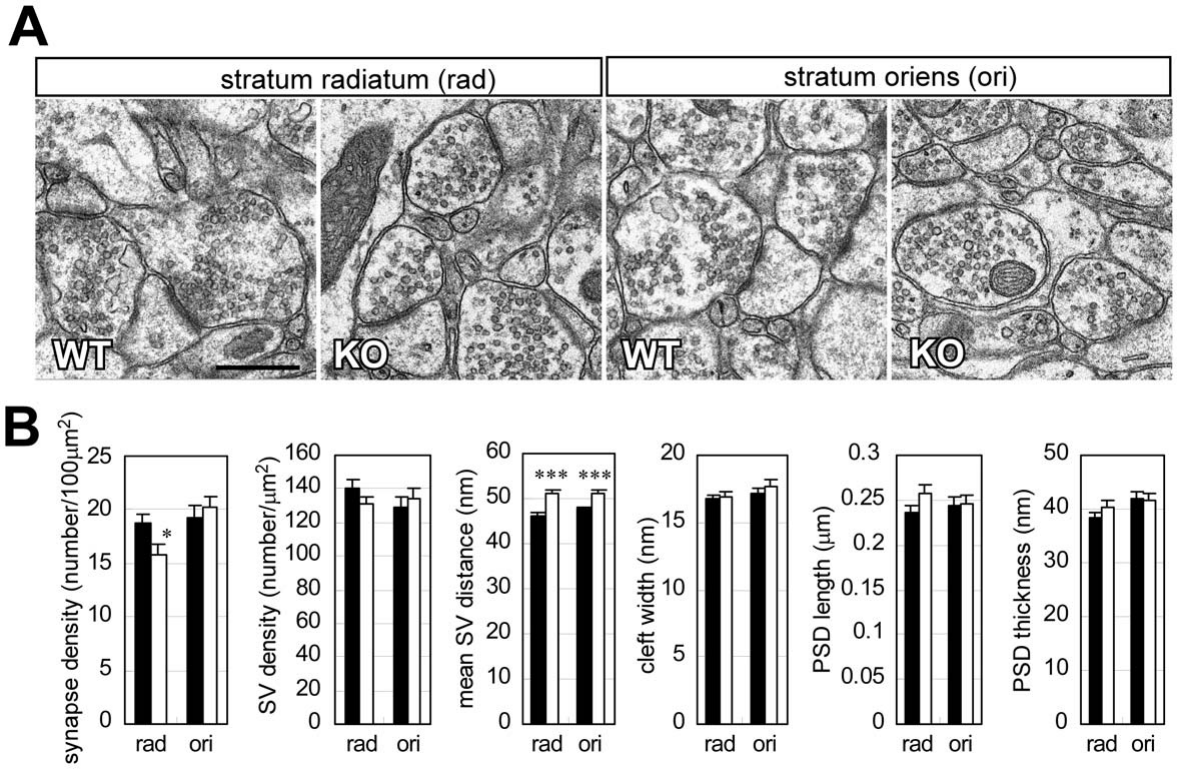
Electron microscopic analysis of hippocampal synapses. (A) Representative images of stratum radiatum and stratum oriens synapses. Scale bar, 500 nm. (B) Quantification of synapse number in 100 µm^2^ of the entire images (synapse density), number of synaptic vesicles in 1 µm^2^ of presynaptic bouton region (SV density), distance between synaptic vesicles (mean SV distance), cleft width, and postsynaptic density (PSD) width (length) and thickness. One hundred and thirty-three synapses from 3 KO mice and 126 synapses from 3 WT mice were analyzed. Black bars, WT; open bars, KO. Values are presented as means ±SEM. * *P*<0.05; *** *P*<0.001.

The above findings led us to further morphologically analyze the *Lrrtm1* KO hippocampus by examining Golgi-stained and electron microscopic images. We found a 7.3% increment in spine length (*P* = 0.0084) (Figure 5D and 5E), a 16% decrement in synaptic density in the stratum radiatum (*P* = 0.032) (Figure 6A and 6B), and increments in the mean inter-vesicular distance in both the stratum radiatum (10%, *P*<0.001) and the stratum oriens (7.4%, *P*<0.001) (Figure 6A and 6B). There were no strong differences in the other structural parameters, including width and density of the dendritic spines (Figure 5D and 5E), postsynaptic density (PSD) length, PSD thickness, and synaptic cleft size (Figure 6A and 6B).

### Difference in effects of MK-801 administration in *Lrrtm1* KO and WT mice

**Figure 7:**
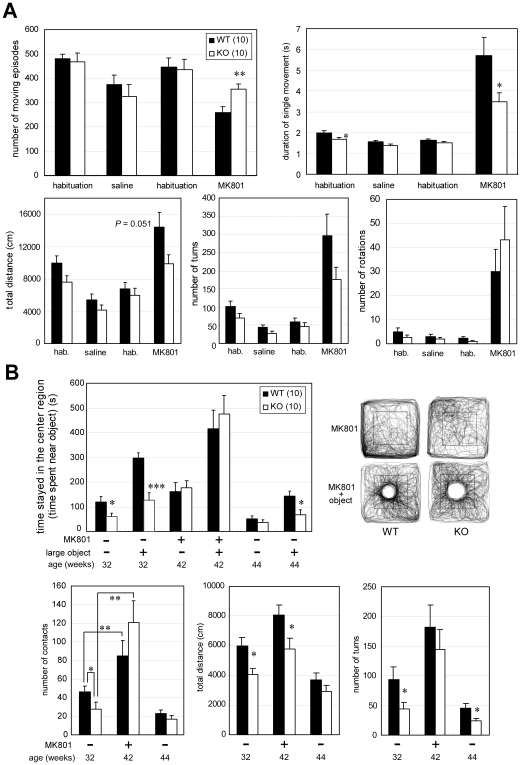
Effects of MK-801 administration on *Lrrtm1* KO behavior in the OF box. (A) Locomotor activities before (habituation) and 10 min after MK-801 treatment or saline treatment were examined in the OF apparatus. Saline injection was done once, followed by MK-801 injection the next day, using the same animals. Number of moving episodes, duration of a single movement, total distance, number of turns, and number of rotations were measured in each 30-min session. (B) Approach to the large object. (*top left*) Time spent in the central area (30% of the total area, indicated as squares in the representative traces at *top right*), which included the large, inanimate object, in the 15-min test period. Compare the traces with those in Figure 3A. As a control, we used the value of the latter half (15 min) of the preceding OF session (large object [–]). (*bottom*) Number of contacts with the large object before, soon after, and 2 weeks after MK-801 treatment. As controls, corresponding values in the large inanimate object approach test (Figure 3A) are indicated (*MK801-*, *32 weeks*). The experiments were done in the same animals at the ages indicated. Values are presented as means ±SEM. * *P*<0.05; ** *P*<0.01, *** *P*<0.001.

The above-mentioned morphological alteration in the hippocampal synapses, together with the spatial memory deficit, raises the possibility of altered hippocampal synaptic transmission. In light of the fact that there is also an altered distribution of VGLUT1-immunopositive signals in *Lrrtm1* KO mice [8], we hypothesized that an altered excitatory synaptic function could underlie some of the behavioral abnormalities in *Lrrtm1* KO mice. To test this hypothesis, we examined the effects of administration of an NMDA receptor blocker, MK-801, on the behavior of KO mice. Ten-month-old mice were injected intraperitoneally with 500 µg/kg of MK-801 or saline during an OF test. Analysis of locomotive behaviors before and after MK-801 administration revealed that, in KO mice, the duration of a single movement was significantly lower (*P* = 0.034) (F(1,18) = 4.5, *P* = 0.049, two-way ANOVA with repeated measures for genotype ×drug interaction), and the number of episodes of movement was significantly higher (*P* = 0.0059) (F(1,18)  = 4.3, *P* = 0.052, two-way ANOVA with repeated measures for genotype × drug interaction) than in WT mice after MK-801 administration (Figure 7A). The total distance moved and the number of turns were non-significantly greater in WT mice than in KO mice after MK-801 administration, whereas the reverse was true for the number of rotations. These changes may reflect the enhanced locomotor activity and stereotypy found with the administration of a similar dose of MK-801 to C57BL/6 mice in previous studies [18], [19]. After the OF test, we also tested the approach to the large object (Figure 7B) that was less frequently contacted by *Lrrtm1* KO mice in the above-described experiments (Figure 3A). After MK-801 administration, the time spent near the object became comparable to that spent by WT mice (Figure 7B, top left), and the number of contacts with the large object by KO mice tended to be even higher than in WT mice (*P* = 0.15) (Figure 7B, bottom left). Two-way ANOVA with repeated measures revealed that there was a significant genotype × MK-801 treatment interaction (F(1,17)  = 5.41, *P* = 0.033). The traces of KO mice during the test were also similar to those of WT mice (Figure 7B, right), in contrast to those without MK-801 administration (Figure 3A). The total distance moved and the number of turns did not show genotype-specific effects of MK-801 (Figure 7B, bottom center and right), suggesting that the increment in approach behavior was not due to an alteration in general locomotor properties. Furthermore, this change was not caused by mere habituation to the object, because a follow-up test performed 2 weeks after MK-801 administration reproduced the changes seen soon after MK-801 treatment (*44 weeks*, Figure 7B). In sum, MK-801 administration induced differences in locomotor activity and attenuated the abnormality in large-object approaching behavior in a genotype-specific manner.

### Effects of antipsychotics and selective serotonin reuptake inhibitor (SSRI)

**Figure 8:**
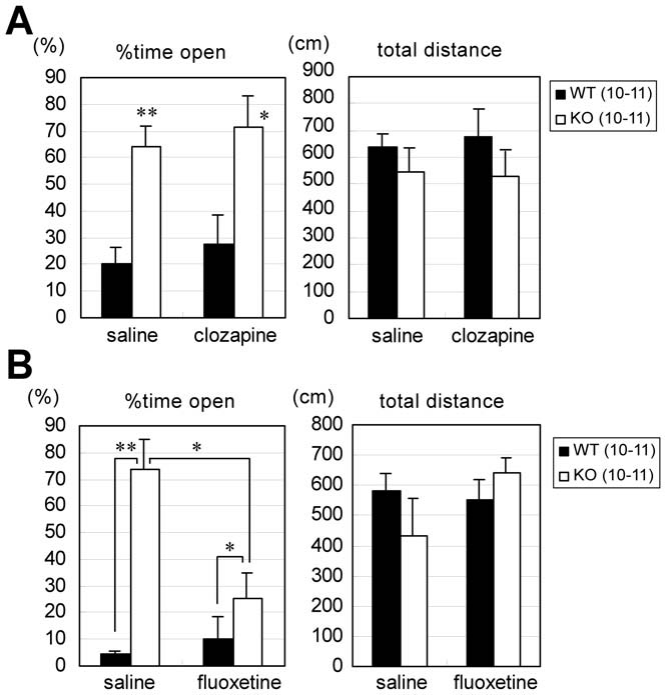
Effects of clozapine or fluoxetine administration on *Lrrtm1* KO behavior in the elevated plus maze test. (A, B) Percentage of time spent in the open arms and total distance traveled in the elevated plus maze test. WT and KO mice were subjected to the test 30 min after intraperitoneal injection of saline, 0.4 mg/kg clozapine, or 10 mg/kg fluoxetine. (A) clozapine treatment. (B) fluoxetine treatment. Values are presented as means ±SEM. * *P*<0.05; ** *P*<0.01 in U-test.

We next evaluated the effect of the antipsychotic clozapine [1], which has been widely used in both clinical and preclinical studies of schizophrenia, on the behavioral abnormalities in *Lrrtm1* KO mice. For the evaluation, we performed EPM tests in which KO mice showed strong reproducible abnormalities in repeated pilot experiments (data not shown). A low dose (0.4 mg/kg) was chosen, because administration of higher doses inhibits all active behavior in mice in the EPM [20]. The time spent in the open arm was not influenced by a single dose of clozapine at 0.4 mg/kg (Figure 8A). Because the impaired behavioral response in a stressful situation looked like a panic-type reaction, we also tested fluoxetine, an SSRI and a first-line drug in panic disorder patients [21]. KO mice given a single dose of 10 mg/kg fluoxetine spent significantly less time in the open arm than did saline-injected KO mice (U = 19, *P* = 0.011), but there was no effect on total distance traveled (Figure 8B). Consistent with the results of a previous study in C57BL/6 mice [22], the time spent in the open arm by WT mice was not significantly affected by 10 mg/kg fluoxetine. Collectively, these experiments revealed that the SSRI effectively rescued the behavioral abnormalities in the EPM test. To determine the effectiveness of antipsychotics on the KO behavioral abnormalities, more systematic analyses with multiple drugs and multiple doses are needed before a conclusion can be drawn.

## Discussion

### *Lrrtm1* KO behavioral abnormalities

*Lrrtm1* KO mice exhibited abnormalities in several behavioral tests. As a frequently observed behavioral abnormality in this study, we emphasize altered behavioral responses to environmental change. The results of the OF, LD, EPM, HB, FC, and MWM tests may be considered in relation to this key concept, as described above. The results of the inanimate object approach experiments may also be considered in this context from a broader perspective, because contact with objects can be regarded as a behavioral response to environmental change. The environmental changes in these tests may have exposed the mice to stressful situations in which they had to evoke behavioral responses. We speculate that Lrrtm1 is necessary for some versatile perception or executive functions required for the appropriate behavioral responses.

We also identified other behavioral abnormalities through our behavioral analysis. One was a social discrimination performance defect in the SD test. Because the test was conducted soon after the training session, the increased response to the familiar mice may indicate impairment of social perception, disturbance of short-term memory formation, or altered emotional status. However, the possibility of the latter two abnormalities may be low, considering that the other behavioral tests did not show abnormalities closely related to these two. The other suggestive abnormality is the spatial memory deficit shown in the MWM test. Although we cannot exclude the influence of altered adaptive response in the training process, the longer distance swum by KO mice was limited to the first day (Figure 4A), and the other parameters—latency in approach to the goal, and no movement time—were not significantly altered in the MWM test (data not shown). We therefore considered that a spatial memory deficit did exist in the *Lrrtm1* KO analysis. On the whole, the behavioral abnormalities in *Lrrtm1* KO mice could be summarized as indicating impaired cognitive function.

### Morphological alteration of hippocampal synapses

The morphological analysis revealed altered synaptic density and morphology in the *Lrrtm1* KO hippocampus. The decrement in synapse density may represent the absence of *Lrrtm1* synaptogenic activity [10]. The longer spines are considered to indicate an abnormality related to postsynaptic differentiation. YFP-tagged Lrrtm1 is known to localize to excitatory synapses in cultured hippocampal neurons and can induce postsynaptic differentiation upon being subjected to an artificial clustering stimulus [10]. On the other hand, the increased inter-synaptic vesicle distances seemed to be consistent with the increment in the size of VGLUT1-immunopositive puncta in the hippocampus of another *Lrrtm1* KO strain [10]; punctum size may be influenced by the distributional area of the synaptic vesicles. Taken together, both the in vivo and the in vitro results indicate that Lrrtm1 exerts important roles in establishing or maintaining synaptic integrity of the hippocampus.

It is interesting that another Lrrtm family, Lrrtm2 [9], can bind neurexin proteins, which are presynaptic transmembrane proteins involved in presynapse differentiation [23]. Considering the fact that the neurexin binding code is conserved in Lrrtm1 [23], Lrrtm1 may be involved in presynapse instruction through an interaction with neurexin-like proteins.

### *Lrrtm1* KO phenotypes and psychiatric disorders

Schizophrenia is characterized by positive symptoms, negative symptoms, and cognitive dysfunction [1], [2]. The impaired cognitive function of *Lrrtm1* KO mice seems to be related to the cognitive dysfunction seen in schizophrenia patients. Furthermore, the increased time spent in the corners of the OF box and the reduction in home-cage activity could be regarded as negative-symptom-related behavioral abnormalities. However, it should also be noted that we did not find any signs suggesting positive-symptom-like abnormalities or sensorimotor gating deficits, which are often reported in mouse models of schizophrenia [24]. The behavioral phenotypes in *Lrrtm1* KO mice thus partly resemble the signs of schizophrenia. Morphologically, the reduction of hippocampal volume is analogous to that seen in first-episode schizophrenia patients [12].

In terms of the pathophysiological basis of the behavioral anomalies seen in the KO mice, alteration in NMDA transmission is suggested by the results of the MK-801 treatment experiment. Because specific malfunction of the glutamate receptor is proposed to be a potential pathogenic mechanism in schizophrenia [25], [26], our results suggest that the involvement of *LRRTM1* dysfunction in schizophrenia needs to be considered. On the other hand, the effectiveness of fluoxetine in the recovery from behavioral response deficit in a stressful situation raises the possibility that a panic-like pathological status exists in *Lrrtm1* KO mice. Although panic disorder is generally considered to fall in the category of anxiety [27], the anxiety-like behaviors in *Lrrtm1* KO mice were not clear. The preference of *Lrrtm1* KO mice to stay in the corners of the OF box suggested enhanced anxiety; however, the LD and EPM tests did not reveal typical traits of enhanced anxiety. In this regard, hasty assumptions should be avoided in correlating the phenotype with the symptoms. It is essential to further clarify the biological role of *Lrrtm1* on the basis of a pharmacobehavioral analysis, longitudinal analysis, and conditional gene targeting. In light of the fact that *LRRTM1* is associated with schizophrenia [7], [8], we suggest that the *Lrrtm1* KO mouse would be useful for further clarifying the involvement of *LRRTM1* in schizophrenia.

## Materials and Methods

### Animals

Mice were maintained by the Laboratory Animal Facility, RIKEN Brain Science Institute. All animal experiments were performed in accordance with the guidelines for animal experimentation at RIKEN. The mice were housed on a 12 h light–dark cycle, with the dark cycle occurring from 8:00 P.M. to 8:00 A.M. The behavior experiments were conducted in a light phase (10:00 AM to 7:00 P.M.). The mice were housed in groups until 1 week before the start of the behavioral experiments, and they were housed singly during the behavioral experiments. In total, 51 pairs of male *Lrrtm1* KO and WT control mice were subjected to the behavioral analysis. The experimental group, the number of KO and WT mice pairs in each group, and the type of behavioral experiment (listed in the order in which the experiments were performed), along with (age [weeks-old] at which the behavioral testing was performed), were as follows: Group 1, 10 pairs, home cage activity (10), OF test (12), LD test (12), EPM test (13), auditory startle response and prepulse inhibition (13), rotarod test (15), MWM test (16), FC test (17); Group 2, 10 pairs, OF test (21), social interaction in the OF (22), marble-burying test (29), OF test (32), resident–intruder test (35), social discrimination test (36), NOR test (37), OF test with MK801 (42), OF test (44); Group 3, 10 pairs, HB test (24), hotplate test (26), tail-flick test (27), MWM test (28), tail suspension test (30), forced swimming test (31); and Group 4, 21 pairs, OF test (14), EPM test (34), EPM test with clozapine (14–34), EPM test with fluoxetine (14–34). To minimize undesirable interexperimental influences, the intervals between the experiments were at least 3 days.

### Generation of *Lrrtm1* KO mice

We generated a conditional knockout of *Lrrtm1*, and the null mutant. To construct the *Lrrtm1* targeting vector, overlapping *Lrrtm1* genomic clones were purchased from BACPAC Resources (Children's Hospital Oakland Research Institute, Oakland, CA, USA). The targeting construct contained the 3.7-kb 5′ and 5.3-kb 3′ homology regions, and the 2.1-kb fragment containing the open reading frame (ORF) of *Lrrtm1* was replaced by an area bounded by two LoxP sequences, together with a phosphoglycerol kinase (PGK) – neomycin-resistance-gene expression cassette flanked by an *FRT* sequence (Figure 1). Embryonic stem cells (EmbryoMax Embryonic Stem Cell Line – Strain C57BL/6, Millipore, Billerica, MA) were electroporated with the targeting construct and selected with G418. Drug-resistant clones were analyzed by Southern blotting. Chimeric mice were generated by injection of the targeted embryonic stem cells into BALB/c blastocysts. To excise the Lrrtm1 protein coding sequence and neo cassette, germline-transmitted mice were first mated with mice transgenic for Cre recombinase under the control of the cytomegalovirus immediate early enhancer – chicken β-actin hybrid (CAG) promoter [28]. Correct excision was confirmed by Southern blot. The resultant allele, which contained a LoxP sequence instead of the 2.1-kb Lrrtm1 ORF-containing region, is called the *Lrrtm1^–^* allele in this study. (*Lrrtm1+/*–, Cre-transgene) mice were backcrossed once to C57BL/6J mice to remove the Cre-transgenes. Lrrtm1 +/– heterozygotes were used to generate *Lrrtm1*–/– mice, which are called *Lrrtm1* KO mice in this study. In all experiments, we used age-matched male *Lrrtm1* KO and WT mice for the analyses. Genotyping was performed by Southern blot or PCR analysis of DNA isolated from tail samples; the PCR primers used were Lr1_5′loxP_F (5′ ATTACCCCGGCTTTGATCTT 3′) and Lr1_3′loxP_R (5′ AGGGAATGATAAAGGGCAGAGA 3′).

### Home-cage activity

Spontaneous activity of mice in their home cages was measured by using a 24-channel Activity Monitoring System (O'Hara, Tokyo, Japan). Cages were individually set into compartments made of stainless-steel in a negative breeding rack (JCL, Tokyo, Japan). A piezoelectric sensor was added to the ceiling of each compartment; it scanned the movements of the mice (approximately 5 times/s). Home-cage activity was measured for 1 week from the afternoon of the day of transfer to the behavioral laboratory (Day 1) until the first day of the next week (Day 8). After the termination of home-cage activity measurement, cages and bedding materials were changed to fresh ones and the mice were maintained in the same type of micro-isolation rack (Allentown Inc., Allentown, NJ, USA) as used in the breeding rooms throughout the behavioral screening.

### OF test

The OF test was performed as previously described [29]. Each mouse was placed in the center of an OF apparatus [50×50×40 (H) cm] illuminated by light-emitting diodes (LEDs; 70 lx at the center of the field) and then allowed to move freely for 15 min. Distance traveled (cm) and time spent (%) in the central area of the field (30% of the field) or in the four corner squares of the 5×5 subdivisions were adopted as indices, and the relevant data were collected every 1 min. Data were collected and analyzed by using Image J OF4 (O'Hara).

### Hole-board test

An OF system made of gray plastic (50×50×40 (H) cm) with four equally separated holes (3 cm diameter with infra-red sensor) on the floor was used (Model ST-1/WII, Muromachi-kikai, Tokyo, Japan). The field was illuminated by fluorescent light (180 lx, at the center of the field), and the level of background noise was approximately 50 dB. The behavior of each mouse was monitored by a CCD camera located about 1.5 m above the field. In the HB test, mice were individually introduced into the center of the field and were then allowed to explore freely for 5 min. Total moving time (s), distance traveled (cm), latency of head-dipping (s), number of head-dips, duration of head-dipping (s), duration of rearing (s), and number of rearings were measured as indices. Data were collected and analyzed by using a CompACT VAS system (Muromachi-Kikai, Tokyo, Japan).

### Light–dark box test

A four-channel LD-box system was added to the same soundproof room as the OF. Each light box was made of white plastic [20×20×20 (H) cm] and illuminated by LEDs (250 lx at the center of the box); a CCD camera was attached to the ceiling. Each dark box was made of black plastic [20×20×20 (H) cm]; an infrared camera was attached to the ceiling. There was a tunnel for transition on the center panel between the light box and dark box (3×5 cm) via a sliding door. In the LD test, mice were individually introduced into the light box, and the door of the tunnel automatically opened immediately after the software detected the mouse. The mice were then allowed to move freely in the LD box for 10 min. Total distance traveled, percentage of time spent in the light box, number of transitions between the light and dark boxes, and the duration of the first latency period before entry to the dark box were measured as indices. Data were collected and analyzed by using Image J LD4 (O'Hara).

### Elevated plus maze test

A single channel of EPM [closed arms: 25×5×15 cm (H); open arms 25×5×0.3 cm (H)) was placed in the same soundproof room that was used for the OF and LD tests. The floor of each arm was made of white plastic, and the wall of the closed arms and the ridge of the open arms were made of clear plastic. The closed arms and open arms were arranged orthogonally 60 cm above the floor. The illuminance at the central platform of the maze (5×5 cm) was 70 lx. In the EPM test, mice were individually placed on the central platform facing an open arm and were then allowed to move freely in the maze for 5 min. Total distance traveled, % of time spent in the open arms, and number of open arm entries as a percentage of the total number of entries were measured as indices. Data were collected and analyzed by using Image J EPM (O'Hara).

### Inanimate object approach tests

This test was performed in the OF apparatus. A mouse was first placed in the OF with 70 lx illuminance for 15 min (habituation session). After the habituation session, the mouse was returned to its home cage and an inanimate object was placed in the center of the field. In the next test session, the mouse was placed again in the OF with the novel object. The large object was prepared by joining two paper cups by their openings (see Figure 3A). Inside the bottom of one cup, a metal block was placed to give stability, and gray monotone and check-patterned printed papers were wrapped around the external surfaces of the cups. Each large object was discarded after use and a new object that had had no contact with the experimental animals was used. The mean time interval between two sessions was 4 min. The total distance traveled and % of time spent in the central area (30% of the field), which included the object and the area around it, were analyzed by using Image J OF4 (O'Hara). Contacts with the novel object were counted on the video records by an observer who was blind to the genotypes. Contact was defined as a forward movement toward the object and subsequent direct contact using the head.

### Novel object recognition test

The experiments were done in accordance with the method of Yoshiike et al. [30]. The test is based on the innate tendency of rodents to differentially explore novel objects over familiar ones. Briefly, the mice were habituated for 15 min to a cage (17 cm×28 cm×12 cm [H]) without bedding materials. After the habituation session, the mice were exposed to two identical small objects for 15 min (training session). Soon after the training session, the mice were presented again with two objects, one used in the training session and a novel object (test session). The used small objects were spherical, conical, cube-shaped, or columnar, made of metal painted black or white in patterns, and generally consistent in their heights and volumes (Figure 3B). The behavior of the mice was video-recorded and the contact with each object was assessed with the naked eye, as in the inanimate object approach test.

### Social discrimination test

This test was performed in the OF test apparatus with 70 lx luminance. The test consisted of a habituation session, first test session, and second test session. Each session continued for 15 min and took place in the following order. In the habituation session, two empty cylindrical wire cages (inner size, 7 cm×15 cm [H]; outer size, 9 cm×16.5 cm [H], with twenty-one 3-mmvertical stainless wires longitudinally and gray polyvinyl discs on the top and the bottom, manufactured by the RIKEN Rapid Engineering Team) were placed in two adjacent corners. In the first test session, a mouse (7-week-old male DBA2, purchased from Nihon SLC, Shizuoka, Japan) that was new to the test mouse was put in one of the two cylindrical cages. In the second test session, another mouse that was also new to the test mouse was put in the remaining cylindrical cage. Between the three sessions there were 4-min intervals, during which the test mouse was returned to its home cage. The three sessions were video-recorded from above, and the times spent in the two corner squares containing the cylinders within the 3- ×3-square subdivision (17.7×17.7 cm square) were measured with Image J OF4 (O'Hara). For the two test sessions, video recording was also done from an obliquely upward position to observe contact between the test mouse and the in-cage mouse. Contact with the in-cage mouse was defined as a forward movement toward the mouse in the cage and subsequent direct contact using the head. The position and posture of the in-cage mouse were observable through the slits of the wires. The contacts were counted on the video records by an observer who was blind to the genotypes. Each in-cage mouse was used once a day; when the habituation session began, the mouse was simultaneously placed in its cylindrical cage on the corners of an OF box that was not being used for the tests. These rules were thought to minimize the difference between the two in-cage mice in the second test sessions in regard to their acclimation to the cylindrical cage and the OF-box environment. After each use, the cylindrical cage was extensively washed with water and rinsed with 90% ethanol, which was then evaporated off, to minimize the effects of remnant materials.

### Morris water maze test

A circular maze made of white plastic (1 m diameter, 30 cm depth) was filled with water to a depth of about 20 cm (22 to 23°C). The water was colored by the addition of white paint to prevent the mice from seeing the platform (20 cm high, 10 cm diameter; 1 cm below the surface of water) or other cues under the water. Some extra-maze landmark cues (i.e. a calendar, a figure, and a plastic box) were visible to the mice in the maze. The movements of the mice in the maze were recorded and analyzed with Image J WM (O'Hara). Mice received six trials ( = 1 session) per day for 4 consecutive days. Each acquisition trial was initiated by placing an individual mouse into the water facing the outer edge of the maze at one of four designated starting points quasi-randomly; the position of the submerged platform remained constant for each mouse throughout the testing. A trial was terminated when the mouse reached the platform, and the latency and distance swum were measured. The cut-off time of the trial was 60 s; mice that did not reach the platform within 60 s were removed from the water and placed on the platform for 30 s before being toweled off and placed back into their home cages. The inter-trial interval was about 6 min. After 4 days of training, a probe test was conducted on day 5. In the probe test, the platform was taken away; each mouse was placed into the water at a point opposite to the target platform and allowed to swim in the maze for 60 s. The distance swum, the number of crossings of the position of the target platform and the other three platforms, and the time spent in each of the four quadrants were measured.

### Classical fear conditioning

This test consisted of three parts: a conditioning trial (Day 1), a context test trial (Day 2), and a cued test trial (Day 3). Fear conditioning was performed in a clear plastic chamber equipped with a stainless-steel grid floor [34×26×30 (H) cm]. A CCD camera was mounted on the ceiling of the chamber and connected to a video monitor and computer. The grid floor was wired to a shock generator. White noise (65 dB) was supplied from a loudspeaker as an auditory cue [i.e. the conditioned stimulus (CS)]. The conditioning trial consisted of a 2-min exploration period followed by two CS–US pairings separated by 1 min. A US (foot-shock: 0.5 mA, 2 s) was administered at the end of the 30-s CS period. Twenty-four hours after the conditioning trial, a context test was performed in the same conditioning chamber for 3 min in the absence of the white noise. A cued test was also performed in an alternative context with distinct cues; the test chamber was different from the conditioning chamber in terms of luminance (about 0 to 1 lx), color (white), floor structure [no grid but with thin bedding material (Alpha-Dri: Shepherd, TN, USA)], and shape (triangular). The cued test was conducted 24 h after the contextual test was finished; it consisted of a 2-min exploration period (no CS) to evaluate nonspecific contextual fear, followed by a 2-min CS period (no foot shock) to evaluate the acquired cued fear. The rate of freezing response (immobility, except for respiration and heartbeat) of mice was measured as an index of fear memory. Data were collected and analyzed with Image J FZ2 (O'Hara).

### Acoustic startle response and prepulse inhibition

For startle response testing, each mouse was put into a small cage (30 or 35 mm diameter, 12 cm long) and the cage was placed on a sensor block in a soundproof chamber [60×50×67 cm (H)]. A dim light was mounted on the ceiling of the soundproof chamber (10 lx at the center of the sensor block), and 65-dB white noise was presented as background noise. In the auditory startle response test, mice were acclimatized to the experimental conditions for 5 min, and then the experimental session began. In the first session, 120-dB startle stimuli (40 ms) were presented to the mice 10 times, with random inter-trial intervals (10 to 20 s). In the second session, startle responses to stimuli at various intensities were assessed. Five white noise stimuli (each 40 ms) at 70 to 120 dB (70, 75, 80, 85, 90, 95, 100, 110, or 120 dB) were presented in quasi-random order and with random inter-trial intervals (10 to 20 s). In the prepulse inhibition session, mice experienced five types of trial: no stimulus; startle stimulus (120 dB, 40 ms) only; prepulse 70 dB (20 ms, lead time 100 ms) and pulse 120 dB; prepulse 75 dB (20 ms, lead time 100 ms) and pulse 120 dB; and prepulse 80 dB (20 ms, lead time 100 ms) and pulse 120 dB. Each trial was performed 10 times in quasi-random order and with random inter-trial intervals (10 to 20 s). In the final session, a 120-dB startle stimuli (40 ms) was presented to the mice 10 times with random inter-trial intervals (10 to 20 s). The total duration of an auditory startle response test was about 35 to 40 min. After each trial, the holding chambers were washed with tap water, wiped with a paper towel, and dried. Apparatuses and software used for the data analysis were commercially available ones (Mouse Startle; O'Hara).

### Effects of MK-801, clozapine, or fluoxetine administration on animal behaviors

MK-801 (Sigma, St. Louis, MO) was dissolved in saline at a concentration of 0.05 mg/ml and administered to mice intraperitoneally at a dose of 0.5 mg/kg. Clozapine (Sigma) was dissolved in small amount of 1N HCl, pH-adjusted to 5 with 1 N NaOH, diluted to 40 µg/ml with saline, and injected at a dose of 0.4 mg/kg. Fluoxetine hydrochloride (Sigma) was dissolved in saline at a concentration of 1 mg/ml and administered at a dose of 10 mg/kg. Control animals were injected with the same volume of saline. MK-801 treated mice were subjected to the OF test. On the first day, after a 30-min habituation period, the mice were given saline, kept in their home cages for 10 min, and then returned to the OF for a 30-min observation session. On the second day, the same procedure was repeated, with substitution of MK-801 solution for the saline. This was followed by the large inanimate object approach test after a 10-min stay in the home cage. Clozapine or fluoxetine were administered to mice intraperitoneally 30 min before EPM tests. The interval between the clozapine and fluoxetine treatments was 6 days. There was no significant interaction between the two drugs in two-way ANOVA for repeated measurement (data not shown). For the stereotypy-like behavior analysis, the turns were calculated from the X,Y coordinates data provided by Image J OF4 (O'Hara) and rotations were counted in video records by the observer blind to genotypes. A turn was defined by crossing the same standard X or Y positions two times within a second. Nine standard positions were set for both X and Y axes to equally divide the OF area.

### Magnetic resonance imaging (MRI) and morphometric analysis

MRI images of adult male mice were acquired by MRI scan using a vertical-bore 9.4-T Bruker AVANCE 400WB imaging spectrometer (Bruker BioSpin, Rheinstetten, Germany). Animals were anesthetized with 3% and 1.5% isoflurane in air (2 L/min flow rate) for induction and maintenance, respectively. MRI images were obtained by using the FISP-3D protocol with Paravision software 5.0 (Bruker BioSpin), with the following parameters: effective echo time  = 4.0 ms, repetition time  = 8.0 ms, flip angle  = 15°, average number  = 5, acquisition matrix  = 256×256×256, and field of view  = 25.6×25.6×25.6 mm. Ten pairs of 36-week-old *Lrrtm1* KO and WT mice were subjected to the analysis. Manual measurements were made of total brain volume, hippocampus volume, and lateral ventricle volume by using Insight ITK-Snap software [4]. Histological examination and immunohistochemical staining were performed as described [31]. Cortical thickness were determined on coronal frozen sections (10 µm, 20 pairs of sections derived from four pairs of *Lrrtm1* KO and WT mice for the prefrontal cortex, motor cortex, and auditory cortex); 50 pairs of sections derived from nine pairs of KO and WT mice were used for the somatosensory cortex.

### Golgi staining

Brains from four pairs of 16-week-old *Lrrtm1* KO and WT mice were Golgi-Cox impregnation-stained by using an FD Rapid GolgiStain kit (FD NeuroTechnologies, Ellicott City, MD, USA). Coronal sections 100 µm thick were prepared. Pyramidal neurons that had clear visible staining from the soma to the distal dendrites were randomly selected, and segments (>20 µm) of distal secondary or tertiary dendrites were scanned (53 segments [2294 spines] from four KO mice, 58 segments [2335 spines] from five WT mice) by using a bright-field microscope (Axioskop 2 Plus, Carl Zeiss Japan, Tokyo, Japan) with a 100× objective. Counting of spines (protrusions) and morphometric analysis of spines were performed as described [5]. Individual spines were manually traced by using NeuroLucida software (MBF Bioscience, Williston, VT, USA), and the maximum length and head width of spines were then measured. Means of these parameters were calculated for each segment and compared between genotypes.

### Electron microscopic analysis

Anesthetized mice (25 weeks old, 3 pairs of *Lrrtm1* KO and WT) were perfused with 2% paraformaldehyde – 2.5% glutaraldehyde in 0.1 M phosphate buffer (pH 7.4). Brains were sectioned at 500 µm, osmicated with 1% OsO_4_ in phosphate buffer, dehydrated through a gradient series of ethanol, and then embedded in epoxy resin (Epon 812, TAAB Laboratories Equipment Ltd., Berkshire, England) by polymerization. Eighty-nanometer-thick ultrathin sections from the hippocampus were cut with an ultramicrotome (Ultracut UCT, Leica Microsystems, Wetzlar, Germany), collected on 200-mesh uncoated copper grids (Maxtaform HF34), and counterstained with uranyl acetate and lead citrate. CA1 stratum radiatum and stratum oriens, in regions about 100 µm apart from the pyramidal cell layer, were examined electron microscopically (Tecnai 12, FEI, Eindhoven, Netherlands). Photographic images were acquired by digital camera (Tem Cam F416, TVIPS, Gauting, Germany) attached to the electron microscope. Mean synaptic densities were calculated by counting asymmetric synapses, which had clear synaptic vesicles with PSD, over an area of more than 10,000 µm^2^ per genotype per region (stratum radiatum and stratum oriens), in 2900× images. For the fine structural analysis, we obtained highly magnified photos (9300×). Every asymmetric synapse with clear presynaptic and postsynaptic membranes was manually analyzed by using NeuroLucida software (MBF Bioscience) for synaptic vesicle density, mean distance between synaptic vesicles, synaptic cleft width, PSD length, and PSD thickness (more than 60 synapses per genotype and per region).

### Statistics

Statistical analyses were conducted by using the SPSS statistical package (ver. 16.0, SPSS Japan Inc., Tokyo, Japan). Parametric data were analyzed by using Student's *t*-test, and non-parametric data were analyzed by using Mann-Whitney's *U*-test. The *P* values refer to the Student's *t*-test unless otherwise noted. Effects of factors were analyzed by using ANOVAs (Uni-ANOVA, two-way ANOVA with *post hoc* tests and General Linear Model [GLM]). Differences were defined as statistically significant when *P*<0.05.

## Supporting Information

Table S1 Summary of Lrrtm1 KO behavioral analysis.
(PDF)
