# Supplementary material for: Impaired Cognitive Function and Altered Hippocampal Synapse Morphology in Mice Lacking Lrrtm1, a Gene Associated with Schizophrenia
Source: PLoS One. 2011 Jul 27;6(7):e22716. doi: 10.1371/journal.pone.0022716 (PMC3144940; doi:10.1371/journal.pone.0022716)
Supplement: Table S1 — Summary of Lrrtm1 KO behavioral analysis. (PDF) [file pone.0022716.s001.pdf]

Table S1 Summary of *Lrrtm1* KO behavioral analysis

test		result	statistical significance and notes
home-cage activity	no. of count by infrared sensor	reduced activity	**, in early dark phase
open field, 70 lx	locomotor	no change	older mice, decreased
	% time in corner	no change	older mice, increased
open field, 250 lx	locomotor	decreased	*
	% time in corner	slightly increased	
light-dark box	% time in light box	no change	
	no. of transitions	decreased	*
	latency entering dark box	increased	*
	total distance	no change	
elevated plus maze	% time in open arm	increased	*
	% entries to open arm	increased	*
	total distance	no change	
hole-board	head-dip latency	increased	**
	total distance	slightly decreased	
	rearing time	no change	
	rearing number	no change	
	head-dip time	no change	
	head-dip number	no change	
inanimate object approach	large object contact time	decreased	*
	small object contact time	no change	
novel object recognition	no. of contacts (training)	increased	***
	no. of contacts (test)	no change	
	preference index	no change	
social interaction in novel environment	contact score	no change	
resident-intruder	investigation	no change	
	attack	slightly decreased	
social recognition of caged mouse	approach to cage	decreased	**
	investigation: novel	no change	

	investigation: familiar	increased	*
water maze	distance swum on day 1	increased	*
	no movement time	no change	
	latency reaching goal	no change	
	probe test of time in quadrant	decreased	*
	probe test of crossing	decreased	*
	rearing after goal reached	increased	
	diving	increased	
fear conditioning - conditioning	freezing response before unconditioned stimulus	increased	*
	freezing response after unconditioned stimulus (tone + footshock)	no change	
fear conditioning - context	freezing response	increased	*
fear conditioning - cue	freezing response before conditioned stimulus	increased	*
	freezing response after conditioned stimulus (tone cue)	increased	*
rotarod	time stayed on rotating rod	no change	
tail suspension	immobility time	no change	
forced swimming	immobility time	no change	
hotplate	latency licking forepaws	no change	
	latency flinching hindpaws	no change	
tail flick	latency removing tail	no change	
acoustic startle response	startle response amplitude	no change	
	prepulse inhibition	no change	
marble burying	% area of marbles buried	no change	
	no. of marbles buried	no change	

Statistical significance indicates the difference between WT and KO, * $P < 0.05$; **$P < 0.01$; ***$P < 0.001$.
